# TWELVE YEARS OF EXPERIENCE USING CHOLECYSTOJEJUNAL BY-PASS FOR PALLIATIVE TREATMENT OF ADVANCED PANCREATIC CANCER

**DOI:** 10.1590/0102-6720201700030009

**Published:** 2017

**Authors:** Marcos Belotto de OLIVEIRA, Bruna do Nascimento SANTOS, André de MORICZ, Adhemar Monteiro PACHECO-JUNIOR, Rodrigo Altenfelder SILVA, Renata D’Alpino PEIXOTO, Tércio De CAMPOS

**Affiliations:** 1 Department of Pancreatic and Bile Duct Surgery, Santa Casa de São Paulo;; 2 Department of Clinical Oncology, Antônio Ermírio de Moraes Oncology Center;; 3 Nove de Julho University, São Paulo, Brazil

**Keywords:** Pancreatic neoplasms, Biliopancreatic diversion, Palliative care

## Abstract

**Background:**

The cholecistojejunal bypass is an important resource to treat obstructive jaundice due to advanced pancreatic cancer.

**Aim:**

To assess the early morbidity and mortality of patients with pancreatic cancer who underwent cholecystojejunal derivation, and to assess the success of this procedure in relieving jaundice.

**Method:**

This retrospective study examined the medical records of patients who underwent surgery. They were categorized into early death and non-early death groups according to case outcome.

**Results:**

51.8% of the patients were male and 48.2% were female. The mean age was 62.3 years. Early mortality was 14.5%, and 10.9% of them experienced surgical complications. The cholecystojejunostomy procedure was effective in 97% of cases. There was a tendency of increased survival in women and patients with preoperative serum total bilirubin levels below 15 mg/dl.

**Conclusion:**

Cholecystojejunal derivation is a good therapeutic option for relieving jaundice in patients with advanced pancreatic cancer, with acceptable rates of morbidity and mortality.

## RESUMO

**Racional:**

A derivação colecistojejunal é um importante recurso para o tratamento de pacientes com icterícia obstrutiva secundária ao câncer de pâncreas avançado.

**Objetivo:**

Avaliar a morbimortalidade precoce dos doentes com câncer de pâncreas submetidos à derivação colecistojejunal, assim como avaliar o alivio da icterícia.

**Método:**

Estudo retrospectivo de prontuários de pacientes que foram operados. Eles foram categorizados de acordo com a resolução dos casos em: morte precoce e sem morte precoce.

**Resultados:**

51,8% dos pacientes eram homens e 48,2% mulheres. A média etária foi de 62,3 anos. A mortalidade precoce foi de 14,5%. 10,9% evoluíram com complicações cirúrgicas. A colecistojejunostomia foi efetiva em 97% dos casos. Houve tendência à maior sobrevida em mulheres e pacientes com níveis séricos de bilirrubina total pré-operatório menor do que 15mg/dl.

**Conclusão:**

A derivação colecistojejunal constitui boa opção terapêutica para alívio da icterícia em pacientes com câncer de pâncreas avançado, apresentando morbimortalidade aceitável.

## INTRODUCTION

Pancreatic cancer is the fourth leading cause of death from malignant neoplasms in the United States^4^, and is three times more common in smokers. The disease is typically classified into two groups: exocrine, which originates in the ductal cells responsible for producing enzymes that aid in digestion, and exocrine, which forms in cells that specialize in producing hormones like insulin. Among exocrine tumors, adenocarcinoma accounts for about 95% of cases, with the majority located in the head of the pancreas. In Brazil, data from the National Cancer Institute (INCA) show that of all types of cancer diagnosed, the incidence of pancreatic cancer has increased to approximately 2% of all cancers and is responsible for more than 8000 cases each year^9^. 

The only curative treatment for pancreatic cancer is surgical resection^5^ ^,^ ^6^. However, fewer than 30% of patients are subjected to this procedure^1^ ^,^ ^7^ ^,^ ^8^ ^,^ ^14^ ^,^ ^15^, because either the disease is initially encountered in a locally advanced state or is metastatic, or because the patient is in poor clinical condition, making large-scale surgical procedures not feasible. Consequently, knowledge of palliative treatment is fundamental; this includes endoscopic, surgical, or radiological procedures or clinical therapeutic measures to relieve pain and obstructive jaundice and to clear duodenal obstruction^17^. 

Palliative treatment of obstructive jaundice stands out because of the frequency of this condition^4^ ^,^ ^17^ ^,^ ^18^ ^,^ ^19^. Because of the morbidity related to obstructive jaundice, particularly the risk of development of cholangitis (inflammation of the biliary duct system, intrahepatic and extra-hepatic inflammation, or both) treatment is essential. The choice of the best therapeutic modality should be based on cost, effectiveness, and ease of execution. 

Although endoscopic or radiological treatments are less invasive, they are not free from complications such as hemorrhage, duodenal perforation, and cholangitis. In the long term, jaundice and cholangitis may return due to migration or occlusion of the stent. As a result, surgical treatment should not be overlooked^2^ ^,^ ^3^ ^,^ ^10^ ^,^ ^12^ ^,^ ^13^ ^,^ ^16^ ^,^ ^20^.

Surgical palliation of obstructive jaundice is obtained through biliodigestive derivations, which can be hepaticojejunal, choledochoduodenal, or cholecystojejunal anastomoses. Cholecystojejunal derivation can contribute to palliative treatment in a greater number of patients because it is a simpler, fast technique that is reproducible in various centers. 

The objective of this study was to assess the morbidity and mortality of patients with pancreatic cancer who underwent cholecystojejunal derivation, as well as the success of this procedure in relieving jaundice.

## METHOD

This study was submitted to and approved by the Research Ethics Committee of Santa Casa de São Paulo.

Data obtained from medical records in the Medical File and Statistical Service (SAME) for patients diagnosed with pancreatic cancer who underwent cholecystojejunal anastomosis procedures at the Pancreas and Bile Duct Group at the Surgery Department of the Irmandade da Santa Casa de Misericórdia de São Paulo Hospital between January 2002 and December 2013 were retrospectively analyzed. 

Inclusion criteria were patients with pancreatic cancer confirmed by biopsy as adenocarcinoma of pancreatic origin for whom resection was not possible (because of either advanced disease or poor clinical condition) and in cases where there was at least 2 cm of space in the main bile duct between the tumor and the implantation of the cystic duct. 

Candidates who underwent cholecystojejunal derivation but did not have adenocarcinoma of the pancreas were excluded. Patients with incomplete medical records were also excluded from the analysis.

The data analyzed by this study were: gender, age, cholangitis (defined as inflammation of the bile duct), serum levels of albumin, creatinine, pre- and post-operatory total bilirubin (TB) and direct bilirubin (BD), glutamic oxalacetic transaminase (sGOT), glutamic pyruvic transaminase (sGPT), prothrombin activity (PA), hemoglobin (Hb), comorbidities, previous surgeries, duration of surgery, need for intraoperative transfusion, length of hospital stay, morbidity as well as median overall survival (OS), cases of early mortality, and improvement rate for jaundice. 

### Statistical analysis

The Kaplan-Meier method was used for statistical analysis of OS, with a log rank test for comparison between groups. P values <0.05 were considered statistically significant. Variables for which univariate analysis reached values of p<0.10 were tested in multivariate analysis using the Cox regression method.

## RESULTS

Were analyzed 55 patients who underwent cholecystojejunal biliodigestive derivation to treat adenocarcinoma of the pancreas. Of these, 51.8% were male and 48.2% were female. Median age was 61 years (variation: 37-93). The main laboratory data obtained are shown in Table 1.

**TABLE 1:** Preoperative characteristics of the participating patients

Parameters	
Male	29/55
Age (years)	62.3
Preoperative cholangitis	Yes=9/55
Preoperative albumin (mg/dl)	3.15+0.73
Preoperative creatinine (mg/dl)	0.74+0.56
Preoperative total bilirubin (mg/dl)	19.65+8.91
Preoperative direct bilirubin (mg/dl)	15.15+7.68
Preoperative sGOT (mg/dl)	169.98+132.6
Preoperative sGPT (mg/dl)	200.92+155.63
Preoperative pre-thrombin activity (%)	77%+14%
Preoperative hemoglobin (mg/dl)	11.28+2.02

**FIGURE 1:**
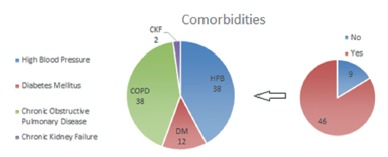
Patient comorbidities according to type and number

**FIGURE 2:**
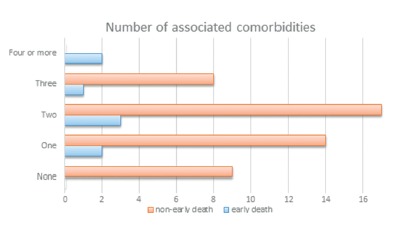
Number of comorbidities per patient, comparing early death and non-early death

Of the patients studied, 16.3% underwent surgery because of cholangitis, and 83.6% had associated comorbidities, most frequently high blood pressure (69%) (Figures 1 and 2). The median duration of surgery was 140 min, ranging from 60 to 300 min. Only two patients required intensive care treatment during the post-operative period. The median hospitalization time after the procedure was four days. 

**FIGURE 3:**
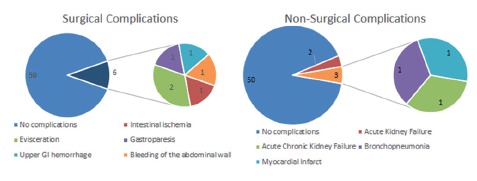
Rate of surgical and non-surgical complications

The early mortality rate (death within 30 days after the procedure) was 14.5%. As for morbidity, 9.0% of patients had no surgical complications while 10.9% developed surgical complications (Figure 3). The cholecystojejunostomy was not successful in 2 cases, 3.3% of patients. In the first case, removal of the biliary obstruction worked until the third month, when the patient become jaundiced again. At this time, hepaticojejunal anastomosis was performed, and the jaundice was resolved for another five months. In the other case the jaundice was not resolved from the beginning; this is probably because the patient had multiple liver metastases, voluminous ascites and carcinomatosis, and the jaundice was not relieved even after transparieto-hepatic drainage. This patient died within 45 days.

**FIGURE 4:**
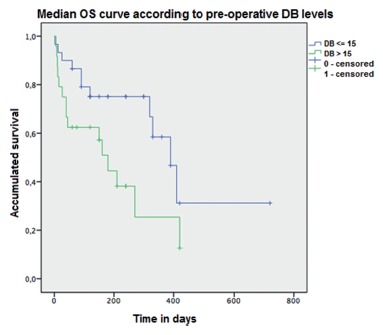
Demonstration of better survival rate in patients with DB levels of <15 mg/dl compared to patients with DB>15 mg/dl (p=0.031)

Median follow-up time was 240 days. Median post-procedure TB and DB levels were 2.2 and 1.4 mg/dl, respectively. Median overall survival (OS) was 320 days (CI 95%: 175-464 days). Patients with DB<15 had median OS superior to patients with DB>15 (307.7 vs. 127.8 days, respectively; p=0.031). There was no difference in median OS according to age (<60 vs. >60 years, p=0.625), Hb values (<11 vs. >11, p=0.938), leukocytes (>8,000 vs. >8,000, p=0.410), cholangitis (yes vs. no, p=0.541), creatinine (<1.0 vs. >1.0, p=0.470), sGOT (<150 vs. >150, p=0.856), sGTP (<150 vs. >150, p=0.434), PA (<70 vs. >70%, p=0.931), albumin (<3.5 vs.>3.5, p=0.238) and comorbidities (yes vs. no, p=0.177). In multivariate analysis (in which only variables with p<0.10 in univariate analysis entered the model), only female remained a factor associated with better OS (hazard ratio 0.62, CI 95% 0.41-0.95, p=0.028). OS was not improved in the group with DB<15 (hazard ratio 0.61, CI 95%, 0.34-1.10, p=0.101, Figure 4). 

Of the eight patients who died less than 30 days post-procedure, two had this outcome less than 24 h after surgery; one of these patients was transferred to our emergency room from another service with cholangitis and septic shock. It was not possible to perform the endoscopic treatment, and the patient died a few hours after the surgery. The second patient presented broncho-aspiration in the early postoperative period which progressed into cardiac arrest. The other six patients died from the following causes: one death by acute vascular abdomen, one on the eighth day due to cholangitis (operated on because of infection), one death from complications due to evisceration and pneumonia, one death due to cardiovascular problems (patient with prior history of myocardial infarction as well as grade IV heart failure and renal failure), and two deaths from upper gastrointestinal bleeding (Table 2).

Of the 47 patients not included in the early death group, 91.5% did not present recurrence of jaundice until the last follow-up or death. Among the four patients who exhibited new episodes of jaundice, this occurred more than six months after the procedure. 

**TABLE 2:** Comparison of the profiles of patients with and without early death

Parameters	Early death group (n=8)	Non-early death group (n=47)	p
Male	6	23	0.2574
Age	65.5+11.8	61.8+11.6	0.4091
Cholangitis	2	7	0.6044
Preoperative hemoglobin (mg/dl)	12.22+1.657	11.12+2.049	0.1577
Preoperative leukocytes (mg/dl)	10.35+4.486	8.72+3.655	0.2669
Total bilirubin (mg/dl)	26.81+13.508	18.44+7.421	0.0126
Preoperative creatinine (mg/dl)	1.05+1.258	0.68+0.328	0.0906
Preoperative sGOT (mg/dl)	158+67.814	172.2+142.120	0.7829
Preoperative sGPT (mg/dl)	176.3+72.360	205.5+167.074	0.6304
Preoperative pre-thrombin activity (%)	84%+12%	76%+14%	0.1342
Preoperative albumin (mg/dl)	3+0.816	3.18+0.721	0.5035
Surgery duration (min)	160+78.010	142.12+54.570	0.4257

## DISCUSSION

Obstructive jaundice is one of the main presentations of pancreatic cancer, especially when this cancer is located in the head of the pancreas^21^. In this series of 185 patients, obstructive jaundice was present in 73% of the 114 patients with tumors located in the pancreatic head, compared with only 11% in cases with tumors located in the body of the pancreas and 0% when tumors were located in the pancreatic tail^21^. Malignant biliary obstruction can lead to harmful consequences such as risk of cholangitis, pruritus, or delayed start of surgical or chemotherapeutic treatment, and can also increase mortality.

Palliative biliary decompression can bring comfort to the patient by improving jaundice and reducing pruritis^3^. Obstructive jaundice can be treated endoscopically, radiologically, or surgically. Endoscopic therapy is a less-invasive technique and has lower mortality rates compared to surgical treatment, but has higher rates of jaundice recurrence^16^. Furthermore, the endoscopic procedure is not always available; many hospitals and even cities do not offer this service 24 h per day, and even places with the endoscopic and radiological equipment often lack the specific materials that are needed to conduct this procedure or the endoscopist is not available. 

In a recent meta-analysis of five randomized studies^1^ that compared surgical decompression with endoscopic stent placement, there was no statistical difference in success rates between the two techniques (relative risk [RR] 0.99, CI 95% 0.93-1.05, p=0.67). Complication and mortality rates also did not differ between the groups (RR 1.54; CI 95% 0.87-2.71, p=0.14). In this meta-analysis, surgical treatment exhibited a mortality rate of approximately 15%, while the mortality rate of the endoscopic treatment was 12% (p=0.40); in our series, the mortality rate was 14.5%. The length of hospital stays evaluated in the meta-analysis was relatively long for both decompression techniques (21.8 days in the surgical group versus 14.6 days in the endoscopic group), while our median hospitalization time was only four days for the cholecystojejunal derivation procedure. Additionally, the recurrence rate for jaundice was an average of nine times lower in patients who underwent surgical treatment compared with endoscopic treatment (RR 0.14; CI 95% 0.03-0.63; p<0.01). However, it should be stressed that of the five studies included in the analysis, four used plastic stents, and in comparison with metal stents plastic is known to have a lower success rate in relieving jaundice^11^. 

Although surgical treatment is more invasive, it alleviates the jaundice for a longer period, so fewer re-admissions are necessary. Furthermore, surgical treatment can be performed in several surgical centers, since complex materials and structures are not required. In patients with adverse clinical conditions, endoscopic treatment should be prioritized, considering its lower morbidity. Nevertheless, even in these severe conditions endoscopic procedures cannot be conducted in countries with limited resources, either because of a lack of around-the-clock availability or lack of materials.

Considering the fact that endoscopic decompression isn’t available in all times, all centers, the high cost and the harder technique, we decided to study the cholecystojejunal bypass because of its ease of execution; it can be performed not only in high-complexity centers, and has acceptable rates of mortality and jaundice resolution according to the international literature. Our study showed that early post-procedure mortality was 14.5%, and that the jaundice recurrence rate in patients who survived more than 30 days after the procedure was only 8.5%, proving the effectiveness of cholecystojejunal derivation.

Although it is relatively easy to perform, some points of caution should be observed when performing a cholecystojejunal derivation. It is essential to inspect the area where the cystic duct will be implanted into the bile duct; this area must be at least 2 cm distant from the tumor. Another fundamental point is to assess whether the wall of the gallbladder wall is in good enough condition to perform the anastomosis. These are the main cautions we observe in our service. 

The relatively long median surgery time (140 min, ranging from 60 to 300) in our study results from the fact that in the same sample we had patients who went to surgery and only underwent cholecystojejunal derivation as well as other patients in whom there was an unsuccessful attempt to do tumor resection, and only the decompression procedure was conducted. 

Our study has some limitations, such as its retrospective nature, the relatively limited number of patients, and the absence of a comparison group subjected to endoscopic biliary decompression. However, the study does show that the cholecystojejunal anastomosis technique can be performed for biliary decompression with high success rates and low morbidity and mortality. In addition, we showed that women and patients with DB levels <15 exhibit better OS after the procedure, which may indicate that earlier clearance of the biliary obstruction is beneficial.

## CONCLUSION

Cholecystojejunal derivation is a good therapeutic option to relieve jaundice in patients with advanced pancreatic cancer which has acceptable morbidity and mortality.
